# Gross and delta efficiencies during uphill running and cycling among elite triathletes

**DOI:** 10.1007/s00421-020-04312-w

**Published:** 2020-04-01

**Authors:** Magnus Carlsson, Viktor Wahrenberg, Marie S. Carlsson, Rasmus Andersson, Tomas Carlsson

**Affiliations:** 1 School of Education, Health and Social Studies, Dalarna University, Högskolegatan 2, 791 88 Falun, Sweden; 2 Swedish Unit for Metrology in Sports, Dalarna University, Högskolegatan 2, 791 88 Falun, Sweden

**Keywords:** Triathlon, Cycling economy, Running economy, Incline, Metabolic rate, Mechanical work rate

## Abstract

**Purpose:**

To investigate the gross efficiency (GE) and delta efficiency (DE) during cycling and running in elite triathletes.

**Methods:**

Five male and five female elite triathletes completed two incremental treadmill tests with an inclination of 2.5° to determine their GE and DE during cycling and running. The speed increments between the 5-min stages were 2.4 and 0.6 km h^−1^ during the cycling and running tests, respectively. For each test, GE was calculated as the ratio between the mechanical work rate (MWR) and the metabolic rate (MR) at an intensity corresponding to a net increase in blood-lactate concentration of 1 mmol l^−1^. DE was calculated by dividing the delta increase in MWR by the delta increase in MR for each test. Pearson correlations and paired-sample *t* tests were used to investigate the relationships and differences, respectively.

**Results:**

There was a correlation between GE_cycle_ and GE_run_ (*r* = 0.66; *P* = 0.038; *R*^*2*^ = 0.44), but the correlation between DE_cycle_ and DE_run_ was not statistically significant (*r* = − 0.045; *P* = 0.90; *R*^*2*^ = 0.0020). There were differences between GE_cycle_ and GE_run_ (*t* = 80.8; *P* < 0.001) as well as between DE_cycle_ and DE_run_ (*t* = 27.8; *P* < 0.001).

**Conclusions:**

Elite triathletes with high GE during running also have high GE during cycling, when exercising at a treadmill inclination of 2.5°. For a moderate uphill incline, elite triathletes are more energy efficient during cycling than during running, independent of work rate.

## Introduction

Triathlon comprises stages of swimming, cycling and running in a sequential order. In a World Cup Olympic-distance competition (i.e. 1.5 km swimming, 40 km cycling and 10 km running), all three stages are important for overall race performance (Landers et al. 2000; Ofoghi et al. 2016). An analysis of the International Triathlon Union’s championship results from 2008 to 2012 revealed that the winners’ mean race times were 1 h 46 min (men) and 1 h 58 min (women) (Ofoghi et al. 2016). Moreover, it was found that elite triathletes’ mean heart rate during an Olympic-distance competition was 92% of their maximal heart rate, which indicates the competition’s high-intensity character (Le Meur et al. 2009).

From a physiological perspective, endurance performance is determined by the sum of the aerobic and anaerobic energy contribution multiplied by gross efficiency (GE) (Joyner and Coyle 2008). In triathlon, performance is mainly determined by maximal oxygen uptake ($\dot{V}{\text{O}}_{{{\text{2max}}}}$), lactate/ventilatory threshold and oxygen uptake kinetics, which together reflect the aerobic energy contribution, and exercise economy (i.e. GE during the specific exercise mode) (Jones and Carter 2000). In line with these findings, lactate-threshold variables and peak oxygen uptake in cycling and running were found to be predictors of Olympic-distance triathlon performance (Miura et al. 1997; Schabort et al. 2000). Hence, the ability to exercise at a lower percentage of $\dot{V}{\text{O}}_{{{\text{2max}}}}$ for a given submaximal workload (i.e. better economy) has been suggested to be of great importance for success in triathlon (Dengel et al. 1989; Sleivert and Rowlands 1996). Accordingly, cycling economy and running economy have been reported to be correlated with performance in triathlon (Miura et al. 1997), and economy of movement has been suggested to be an important determinant of triathlon performance (Dengel et al. 1989; Tucker and Tucker 2013).

The economy of movement is reflected by the functioning of the cardiorespiratory, metabolic, neuromuscular and biomechanical systems (Barnes and Kilding 2015; Ettema and Lorås 2009). In line with this concept, it has been suggested that running economy is related to factors such as muscle morphology, elastic elements and joint mechanics (Barnes and Kilding 2015; Joyner and Coyle 2008; Lacour and Bourdin 2015). In cycling, mechanisms such as muscle-fibre-type transformation, changes in muscle-fibre-shortening velocities, changes within the mitochondria and biomechanical factors have been proposed to be related to improved cycling economy (Coyle et al. 1991; Hopker et al. 2009).

There are several ways to express cycling efficiency. Two of these measures of efficiency are based on the relationship between the work performed and the energy expenditure; GE is the ratio between the mechanical work rate (MWR) and the metabolic rate (MR) (i.e. GE = MWR/MR), whereas delta efficiency (DE) is the ratio between the delta increase in MWR and the delta increase in MR (i.e. DE = ΔMWR/ΔMR). In cycling, GE varies between approximately 18 and 23% in different individuals (Coyle et al. 1992), and the corresponding range in DE is approximately 18–27% (Coyle et al. 1992; Ettema and Lorås 2009). The DE is usually somewhat higher than GE because the basal metabolic rate and metabolic cost of zero-load exercise are excluded from DE calculations.

Running economy is often measured as oxygen uptake at a given submaximal running speed (e.g. 16 km h^−1^) while running on a level treadmill, where a better running economy is indicated by a lower oxygen consumption (Barnes and Kilding 2015). During level treadmill running, zero external work is performed against gravity, frictional forces or air resistance; hence, it is not appropriate to express running economy as GE or DE using a treadmill inclination of 0°. Previously, it has been found that GE during running increases with steeper inclines (Minetti et al. 2002), which emphasize the importance of taking the incline into account when running efficiency is evaluated.

A recent study investigated the relationship between triathletes’ energy expenditure during level running at 12 km h^−1^ and during ergometer cycling at a power output of 200 W, and no significant correlation was found between the gross metabolic rates (Swinnen et al. 2018). Other studies have compared DE during running and cycling using different methods to apply external loads (e.g. running up different inclines, applying impeding horizontal forces during level treadmill running and treadmill cycling on a tricycle), but the relationship between running and cycling DE was not investigated in either study (Bijker et al. 2001, 2002).

To the best of our knowledge, no previous study has used a fixed treadmill inclination to investigate elite triathletes’ running and cycling efficiencies. The purpose of this study was to investigate gross efficiency and delta efficiency during cycling and running in elite triathletes.

## Methods

### Participants

Five male (age: 24 ± 5 years, stature: 181 ± 4 cm, and body mass: 73 ± 4 kg) and five female (age: 22 ± 6 years, stature: 169 ± 8 cm, and body mass: 64 ± 9 kg) elite triathletes volunteered to participate in the study and completed the GE and DE tests. During a 5-year period, all ten triathletes had been in the top 8 in the Swedish championships; seven of the participants had at least one podium finish, and two participants had previously won the Swedish championships in triathlon.

### Testing procedures

The participants were instructed to only perform light training on the 2 days preceding their scheduled test days, to be well hydrated, to refrain from alcohol (24 h) and caffeine (12 h) and to avoid eating within 2 h prior to testing. On the day of the tests, the participants completed a health-status questionnaire, and thereafter, the participant’s stature (Harpenden Stadiometer, Holtain Limited, Crymych, Great Britain) and body mass (Midrics 2, Sartorius AG, Goettingen, Germany) were measured. Additionally, the mass of the equipment the participant used in the cycling test (i.e. bicycle, cycling shoes, helmet and harness) and running test (i.e. running shoes and harness) were weighed.

The cycling and running tests were performed on a motor-driven treadmill (Saturn 450/300rs, h/p/cosmos sports & medical GmbH, Nussdorf-Traunstein, Germany). Throughout the tests, expired air was continuously analysed using a metabolic cart in mixing-chamber mode (Jaeger Oxycon Pro, Erich Jaeger Gmbh, Hoechberg, Germany). The metabolic cart was calibrated according to the specifications of the manufacturer before each test, and at the start of each new 5-min stage, a ‘zeroing’ of the O_2_ and CO_2_ sensors was performed. After the warm-ups and after each stage was completed, capillary-blood samples were collected from a fingertip and thereafter analysed to determine blood-lactate concentrations (Biosen 5140, EKF-diagnostic GmbH, Barleben, Germany).

### Cycling test

Prior to the cycling test, the participants performed a standardized warm-up. The 7.5-min warm-up started with 5 min at a treadmill inclination of 1° and treadmill speed of 5.56 m s^−1^ (20 km h^−1^) for the men and 5.00 m s^−1^ (18 km h^−1^) for the women, which was followed by 2.5 min at the initial work intensity of the cycling test (i.e. inclination, 2.5°; speed, 4.56 m s^−1^ (16.4 km h^−1^) (men) or 3.22 m s^−1^ (11.6 km h^−1^) (women)). After the warm-up was completed, a capillary-blood sample was collected, and thereafter the rolling-resistance coefficient of the participant’s bicycle was determined using a previously described method (Carlsson et al. 2016). In brief, the treadmill speed was set at 5.56 m s^−1^ (20 km h^−1^), with the rider facing downhill, and the treadmill’s negative inclination was then adjusted until the participant sitting on the bicycle (without pedalling) did not move in either the backward or forward direction on the treadmill. Based on the equilibrium inclination, the bicycle’s rolling-resistance coefficient (*μ*) was calculated from the formula *μ* = *m*_tot_ · *g* · sin *α*/*m*_tot_ · *g* · cos *α*, where *m*_tot_ is the mass of the participant, including the mass of the equipment (kg), *g* is the acceleration due to gravity (9.82 m s^−2^ at the location of the sport-science laboratory) and *α* is the treadmill inclination (°).

Throughout the cycling test, the treadmill inclination was 2.5°, and the participants were permitted to use a self-chosen cadence. For each of the subsequent stages, the speed was increased by 0.67 m s^−1^ (2.4 km h^−1^). Each stage lasted for 5 min, and the mean oxygen uptake ($\dot{V}{\text{O}}_{{{\text{2mean}}}}$) and mean respiratory exchange ratio (RER_mean_) during the last 2 min of the stages were used for calculation of gross efficiency (GE_cycle_) and delta efficiency (DE_cycle_) during cycling. The stages were separated by a 1-min pause to collect a capillary-blood sample, and the participants rated their perceived exertion (RPE) on a scale of 6–20 (Borg 1970). The pre-determined criteria for permitting the participants to commence another stage were as follows: the participant’s RPE had to be lower than 17 (“Very hard”), and the previous stage’s RER had to be lower than 1.0.

### Running test

To minimize the influence of the cycling test on the subsequent running test, the running test was initiated 60 min after the completion of the cycling test. It has previously been reported that approximately 30 min of passive recovery is sufficient to reduce the blood-lactate concentration from 3.9 ± 0.3 mmol l^−1^ to baseline values (1.0 ± 0.1 mmol l^−1^) in moderately trained adults (Menzies et al. 2010); hence, the time for the elite triathletes to recover after the submaximal cycling test was considered to be sufficient. The participants were permitted to use a self-chosen stride frequency throughout the test. Prior to the start of the running test, the participants performed a 5-min warm-up at a treadmill inclination of 2.5°, and the treadmill speeds were 10.0 km h^−1^ and 8.2 km h^−1^ for the men and women, respectively. After the warm-up was completed, a capillary-blood sample was collected. Thereafter, the running test was initiated with the same intensities as in the warm-up; it consisted of 5-min stages with a 1-min pause between stages to collect a capillary-blood sample, and to have the participants rate their perceived exertion. Throughout the test, the treadmill inclination was fixed at 2.5°, and the treadmill speed increment was 0.6 km h^−1^ between stages. The criteria for permitting the participants to commence another stage were the same as those for the cycling test. The $\dot{V}{\text{O}}_{{{\text{2mean}}}}$ and RER_mean_ during the last 2 min of the stages were used for calculation of the gross efficiency (GE_run_) and delta efficiency (DE_run_) during running.

### Calculation of delta efficiency

For each completed stage in both tests, i.e. when RER was < 1.0 and blood-lactate concentration was < 4.0 mmol l^−1^, the mechanical work rate (MWR) and metabolic rate (MR) were calculated. The MWR (W) during the cycling test was the sum of the work against gravity and the work related to overcoming the rolling resistance of the bicycle: MWR = (*m*_tot_ · *g* · sin *α* · *v* + *m*_tot_ · *g* · cos *α* · *μ* · *v*), where *v* is the treadmill speed (m s^−1^). In the running test, only the component related to work performed against gravity was included in the MWR calculation. The MR (W) was based on the participant’s $\dot{V}{\text{O}}_{{{\text{2mean}}}}$ (l s^−1^) and RER_mean_: MR = *k*_1_ · $\dot{V}{\text{O}}_{{{\text{2mean}}}}$ · *k*_2_, where *k*_1_ is 3.815 + 1.232 · RER_mean_ (Lusk 1928) and *k*_*2*_ is 4186 and converts kcal to J. Linear regression was used to determine the relationship between MWR and MR for each participant. Based on the relationship, DE_cycle_ and DE_run_ were calculated by dividing the delta increase in MWR by the delta increase in MR for each test.

### Calculation of gross efficiency

For both tests, the GE was calculated as the ratio between the MWR and MR during the last 2 min of each stage. To make an adequate comparison between GE_cycle_ and GE_run_, the MWR when the blood-lactate concentration had increased 1 mmol l^−1^ above the lowest measured value (LT) was used. To establish the work rate at the LT, a third-order polynomial equation was fitted to each of the participant’s obtained MWR/blood-lactate concentration combinations, i.e. it was calculated even for those stages that resulted in lactate values exceeding 4 mmol l^−1^. The polynomial equation was then used to calculate the MWR at the LT. Thereafter, linear regression was used to determine the relationship between MWR and GE for each participant. Based on the linear equation and the participant’s MWR at the LT, the GE at the LT was calculated.

### Statistical analyses

The test results are presented as the mean and standard deviation (SD). The agreement of test variables with a normal distribution was assessed with the Shapiro–Wilk test. Pearson’s product–moment correlation coefficient (*r*) test was used to investigate the relationship between GE_cycle_ and GE_run_ as well as between DE_cycle_ and DE_run_. The guidelines for the interpretation of the strength of the correlation are as follows: small correlation for 0.1 ≤|*r*|< 0.3, moderate correlation for 0.3 ≤|*r*|< 0.5, and large correlation for |*r*|≥ 0.5 (Cohen 1988). Paired-samples *t* tests were used to investigate differences between GE_cycle_ and GE_run_ as well as between DE_cycle_ and DE_run_. The Cohen's effect-size criteria were used to interpret the magnitude of the effect size (*η*^2^) and to enable making more informative inferences from the results. The substantial effects were divided into more fine-graded magnitudes as follows: small effect for 0.01 ≤ *η*^2^ < 0.06, moderate effect for 0.06 ≤ *η*^2^ < 0.14, and large effect for *η*^2^ ≥ 0.14 (Cohen 1988). All statistical analyses were assumed to be significant at an alpha level of 0.05. The statistical analyses were conducted using the IBM SPSS Statistics software, Version 25 (IBM Corporation, Armonk, NY, USA).

## Results

The test results of the cycling and running test are presented in Tables 1 and 2, respectively. The mass of the equipment was 9.6 ± 0.5 kg during the cycling test and 0.9 ± 0.2 kg during the running test. The bicycles’ *μ* was determined to 0.0042 ± 0.0006 N N^−1^. The intercepts for the relationship between MWR and oxygen uptake were 0.44 ± 0.12 l min^−1^ and 0.28 ± 0.20 l min^−1^ for the cycling and running test, respectively. The blood-lactate concentrations at LT were 1.9 ± 0.2 mmol l^−1^ for the cycling test and 2.2 ± 0.3 mmol l^−1^ for the running test. The maximum blood-lactate concentration after each test was 4.3 ± 1.1 mmol l^−1^ and 4.0 ± 1.8 mmol l^−1^ for the cycling and running test, respectively.

**Table 1:** Test results from the cycling test

Stage	$\dot{V}{\text{O}}_{{{\text{2mean}}}}$	RER	MR	MWR	GE
1	2.16 ± 0.50	0.80 ± 0.03	726 ± 170	144 ± 37	19.7 ± 0.8
2	2.42 ± 0.51	0.84 ± 0.03	820 ± 175	168 ± 39	20.4 ± 0.7
3	2.72 ± 0.55	0.85 ± 0.03	921 ± 187	193 ± 41	20.9 ± 0.7
4	3.03 ± 0.60	0.86 ± 0.03	1032 ± 206	217 ± 44	21.0 ± 0.6
5	3.32 ± 0.63	0.90 ± 0.02	1140 ± 216	242 ± 46	21.2 ± 0.7
6	3.61 ± 0.67	0.93 ± 0.02	1250 ± 232	269 ± 47	21.5 ± 0.7
7	N/A	N/A	N/A	N/A	N/A
LT	3.14 ± 0.62	0.87 ± 0.04	1069 ± 209	226 ± 45	21.1 ± 0.7

All values are presented as mean ± standard deviation

$\dot{V}{\text{O}}_{{{\text{2mean}}}}$ mean oxygen uptake (l min^−1^), *RER* respiratory exchange ratio (l l^−1^), *MR* metabolic rate (W), *MWR* mechanical work rate (W), *GE* gross efficiency (%), *LT* the MWR at which the blood-lactate concentration increased 1 mmol l^−1^ above the lowest measured value. All ten participants completed stage 1–5. Stages 6 and 7 were completed by eight and zero participants, respectively, *N/A* not applicable

**Table 2:** Test results from the running test

Stage	$\dot{V}{\text{O}}_{{{\text{2mean}}}}$	RER	MR	MWR	GE
1	2.73 ± 0.47	0.83 ± 0.03	923 ± 157	76 ± 15	8.2 ± 0.5
2	2.89 ± 0.49	0.84 ± 0.03	977 ± 164	81 ± 15	8.2 ± 0.6
3	3.03 ± 0.51	0.85 ± 0.03	1027 ± 172	86 ± 16	8.3 ± 0.6
4	3.23 ± 0.54	0.86 ± 0.04	1097 ± 180	90 ± 17	8.2 ± 0.6
5	3.38 ± 0.53	0.87 ± 0.04	1152 ± 177	95 ± 17	8.3 ± 0.5
6	3.57 ± 0.63	0.89 ± 0.05	1222 ± 210	101 ± 19	8.2 ± 0.5
7	3.79 ± 0.52	0.89 ± 0.05	1298 ± 171	112 ± 15	8.6 ± 0.2
LT	3.41 ± 0.69	0.88 ± 0.04	1165 ± 227	96 ± 22	8.2 ± 0.5

All values are presented as mean ± standard deviation

$\dot{V}{\text{O}}_{{{\text{2mean}}}}$ mean oxygen uptake (l min^−1^), *RER* respiratory exchange ratio (l l^−1^), *MR* metabolic rate (W), *MWR* mechanical work rate (W), *GE* gross efficiency (%), *LT* the MWR at which the blood-lactate concentration increased 1 mmol l^−1^ above the lowest measured value. All ten participants completed stage 1–5. Stage 6 and 7 was completed by 9 and 5 participants, respectively.

**Fig. 1:**
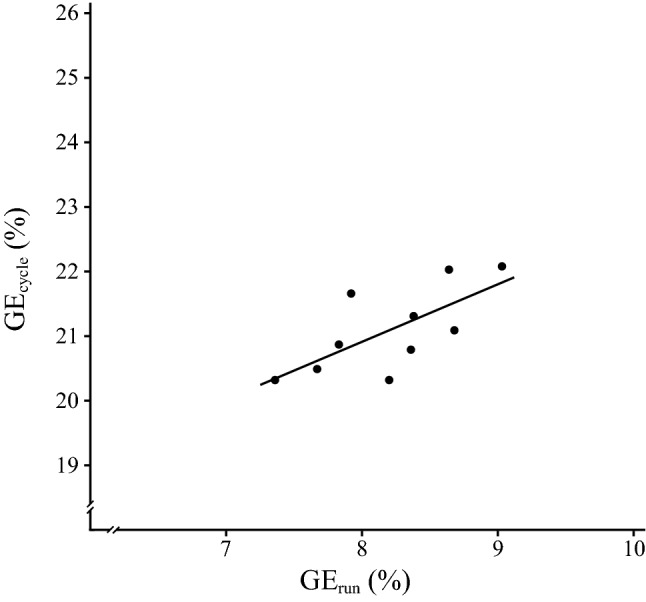
Significant relationship between gross efficiency during running (GE_run_) and gross efficiency during cycling (GE_cycle_) (*P* < 0.05)

**Fig. 2:**
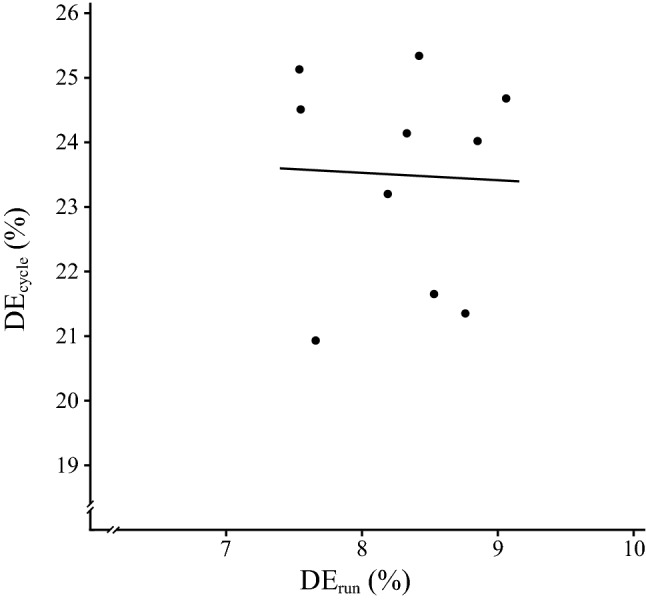
No significant relationship between delta efficiency during running (DE_run_) and delta efficiency during cycling (DE_cycle_) (*P* > 0.05)

The test results in the DE tests were DE_cycle_ = 23.5 ± 1.6% and DE_run_ = 8.3 ± 0.5%. All test variables were normally distributed (all *P* > 0.05). There was a correlation between GE_cycle_ and GE_run_ (*r* = 0.66; *P* = 0.038; *R*^2^ = 0.44) (Fig. 1), and the participants’ sex was not a contributing factor (*P* = 0.79). There was a significant relationship between oxygen uptake values during cycling and running at LT (*r* = 0.95; *P* < 0.001; *R*^2^ = 0.90). No significant relationship was found between DE_cycle_ and DE_run_ (*r* = − 0.045; *P* = 0.90; *R*^2^ = 0.0020) (Fig. 2), and the participants’ sex was not a contributing factor (*P* = 0.38).

**Fig. 3:**
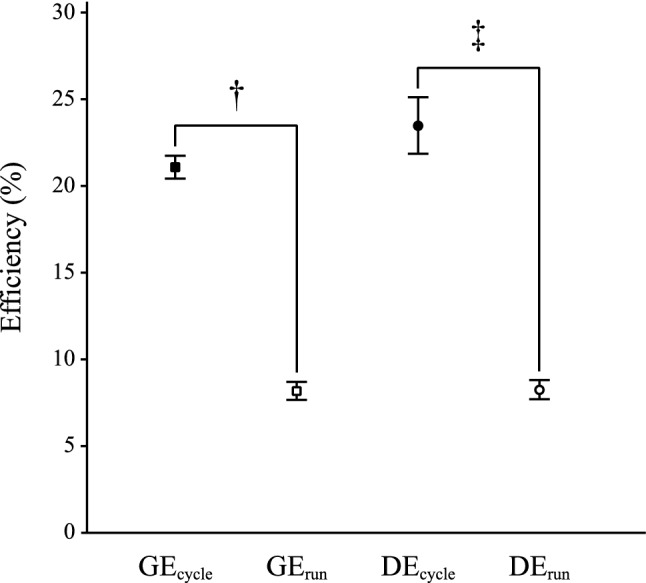
Significant differences between gross efficiency during running (GE_run_) and cycling (GE_cycle_) is reported as ^†^*P* < 0.001, and between delta efficiency during running (DE_run_) and cycling (DE_cycle_) is reported as ^‡^*P* < 0.001. Squares and circles represent mean values, and error bars represent ± 1 standard deviation

There were differences between GE_cycle_ and GE_run_ (*t* = 80.8; *P* < 0.001; *η*^2^ = 0.99) as well as between DE_cycle_ and DE_run_ (*t* = 27.8; *P* < 0.001; *η*^2^ = 0.98) (Fig. 3). Moreover, there was a difference between GE_cycle_ and DE_cycle_ (*t* = − 5.85; *P* < 0.001; *η*^2^ = 0.79); however, no difference was found between GE_run_ and DE_run_ (*t* = − 0.40; *P* = 0.70; *η*^2^ = 0.018).

## Discussion

The results of this study demonstrate that there is a large correlation between elite triathletes’ GE during running and cycling on a moderate uphill incline. However, no correlation was found between DE during running and cycling. The results reveal that GE and DE differ between cycling and running, with large effect sizes, where cycling is more energy efficient than running on a moderate uphill incline.

The finding that GE_run_ and GE_cycle_ are strongly correlated (Fig. 1) is consistent with results of previous studies that reported a significant positive correlation between cyclists’ running economy and cycling economy when the economy of movement was measured during level running and ergometer cycling, respectively (Lundby et al. 2017; Swinnen et al. 2018). Moreover, in the current study, there was a significant correlation between gross metabolic rates (i.e. oxygen uptake) during cycling and running. This result contradicts the result from recent study that found no significant relationship between the gross metabolic rates during running and cycling for nine sub-elite triathletes (Swinnen et al. 2018); however, the relationship was close to significance (*P* = 0.053).

The large correlation between exercise-mode efficiencies found in the current study indicates that an elite triathlete with a high GE_run_ also has a high GE_cycle_. This result contradicts previous findings that the efficiency in one exercise mode does not predict the efficiency in other exercise modes (Daniels et al. 1984). However, it should be noted that in the study by Daniels et al. (1984), trained runners were tested in exercise modes outside their specific sport (i.e. bench stepping, arm cranking, graded walking and cycling) in addition to running. In the case of the participants in the current study, one can assume that to become a national level elite triathlete it is important to be efficient at all three disciplines in triathlon, which partly could explain the interrelationship of GE_run_ and GE_cycle_.

The exercise efficiency is determined by the cardiorespiratory, metabolic, neuromuscular and biomechanical efficiencies (Barnes and Kilding 2015). The cardiorespiratory and metabolic efficiencies reflect the delivery of oxygen to the force-producing muscles and the adenosine triphosphate re-synthesis therein (Barnes and Kilding 2015; Saunders et al. 2004). The neuromuscular and biomechanical efficiencies reflect the interactions between the neural and musculoskeletal systems as well as the efficiency of converting produced power to forward propulsion (Anderson 1996; Barnes and Kilding 2015). The energy expenditure during cycling and running is related to the increase in potential energy during the pedal cycle/stride cycle (i.e. the raising of centre of mass vertically during the pedal cycle/stride cycle), the translational kinetic energy (i.e. the braking and propelling of the body mass in the forward direction parallel to the surface) and the rotational kinetic energy (i.e. the swinging of the legs and arms) as well as the maintenance of balance and energy cost of supporting body weight (Bergh 1987; Hoogkamer et al. 2014). Hence, a triathlete’s GE is determined by these four underlying efficiencies (i.e. cardiorespiratory, metabolic, neuromuscular and biomechanical efficiency) and at least one of these underlying efficiencies is significantly higher for a ‘more efficient’ triathlete compared to their ‘less efficient’ counterpart.

In the current study, it was found that GE_cycle_ was significantly lower than DE_cycle_ (Fig. 3), and the difference was associated with a large effect size. This difference is to a large extent explained by the influence of baseline energy expenditure in the GE calculations, which previously has suggested being an artefact (Gaesser and Brooks 1975). The relative contribution of the baseline energy expenditure in the GE calculations decreases gradually with increasing work intensity; hence, it should be expected that the GE during cycling is related to work intensity. This is in line with a previous study that reported a positive relationship between cyclists’ GE and crank inertial load (Bertucci et al. 2012). In the running test, there was no difference between GE_run_ and DE_run_, which means that triathletes’ GE during moderate uphill running does not change significantly with increasing work rate. Calculations of GE during uphill running, based on reported values for running speed and treadmill inclination as well as the mean values of body mass and oxygen uptake (Hoogkamer et al. 2014), showed that GE was independent of running speed (2–3 m s^−1^); GE was calculated to be approximately 7% at a 2° incline and 10% at a 3° incline, which are in good agreement with the results in the current study.

When comparing GE_run_ with GE_cycle_ at the same external work rate and treadmill incline, cycling was shown to be more energy efficient than running (21.1% versus 8.2%, respectively) (Fig. 3), despite the limitation that the equation for calculating MWR during cycling does not account for the work done to overcome the friction of the drivetrain of the bicycle. Hence, the GE_cycle_ is therefore somewhat underestimated. At an equivalent metabolic energy expenditure rate, the mechanical power output during cycling was approximately 2.5 times higher than during running. Hence, based on the previously presented equation that endurance performance is equal to the sum of aerobic and anaerobic energy contributions multiplied by GE (Joyner and Coyle 2008), it can be concluded that the cycling speed is much higher than the running speed for a moderate uphill incline at the same work intensity as a consequence of the higher GE during cycling. The GE difference between cycling and running is to a large extent explained by differences in the factors related to force generation to support body weight, an increase in potential energy during the pedal cycle/stride cycle and to translational kinetic energy. Running entails a considerably higher vertical raising of the centre of mass (~ 8–10 cm) (Cavagna et al. 2005; Tartaruga et al. 2012) compared to cycling, where the raising of the centre of mass is minimal during pedalling (Connolly 2016). The importance of having a relatively low vertical centre-of-mass displacement during running is indicated by a lower energy cost and thus better running economy (Folland et al. 2017). Moreover, the stride cycle during running implies a deceleration at the foot plant, followed by an acceleration of body mass at push-off (Hamner et al. 2010). Based on fundamental physics, these deceleration/acceleration phases are associated with a significant energy cost; however, the relative energy-cost contribution related to translational kinetic energy decreases during uphill running, because on inclines the braking forces at the foot plant decrease (Gottschall and Kram 2005). In cycling, the fluctuation in speed during the pedal cycle is lower than in running due to the continuous supply of power (Fintelman et al. 2016; van Ingen Schenau et al. 1990). The described energy-expenditure differences between the exercise modes result in a reduced biomechanical efficiency during running compared to cycling, which is the major factor explaining the lower GE during running.

The corresponding reasoning could be applied to understand the difference, and large effect size, between DE_run_ and DE_cycle_ (Fig. 3), because DE reflects how much a triathlete needs to increase his/her MR for an increment in MWR. Hence, the enhanced biomechanical efficiency during cycling compared to running is reflected in a higher DE_cycle_ than DE_run_. This finding contradicts results from previous studies that reported higher DE during running (~ 44%) compared to cycling (~ 25%), where DE was derived from tests using a constant running speed with an incremental increase in treadmill inclination and stationary ergometer cycling (Bijker et al. 2001, 2002). Previously it was reported that the metabolic cost of running parallel to the running surface decreases with incline, whereas the efficiency of producing mechanical power to lift the centre of mass vertically is constant and independent of incline and running speed (Hoogkamer et al. 2014). Hence, the methodological differences (i.e. constant speed and incremental increase in incline vs. incremental increase in speed and constant incline) could to a large extent explain the contradicting DE values during running.

The correlation analysis showed that participants’ sex was not a contributing factor to the relationship between GE_cycle_ and GE_run_. This result is in line with previously reported results where GE during cycling did not differ between male and female competitive cyclists when the sexes were compared at the same relative intensity (Hopker et al. 2010). Moreover, in a recently published review investigating factors affecting the energy cost of running, it was concluded that men and women with the same body mass have similar running economies (Lacour and Bourdin 2015). However, because of the low number of participants in the current study further research is warranted to investigate potential sex differences in GE for elite triathletes during cycling and running.

## Conclusions

The results show that elite triathletes with high GE during running also have a high GE during cycling, when exercising at a treadmill inclination of 2.5°. However, a triathlete’s relative efficiency in attaining an increased power output in terms of DE is not transferable between the two exercise modes. In a moderate uphill incline, elite triathletes are more energy efficient during cycling than running, independent of work rate.

## Abbreviations

*α*: Treadmill inclination
DE: Delta efficiency
DE_cycle_: Delta efficiency during uphill cycling
DE_run_: Delta efficiency during uphill running
ΔMR: Change in metabolic rate
ΔMWR: Change in mechanical work rate
*g*: Gravitational acceleration
GE: Gross efficiency
GE_cycle_: Gross efficiency during uphill cycling at the lactate threshold
GE_run_: Gross efficiency during uphill running at the lactate threshold
LT: Lactate threshold, i.e. the mechanical work rate at which the blood-lactate concentration increased 1 mmol l^−^^1^ above the lowest measured value
*m*_tot_: Total mass of participant and equipment
MR: Metabolic rate
MWR: Mechanical work rate
RER_mean_: Mean respiratory exchange ratio
*μ*: Rolling-resistance coefficient of the bicycle
$\dot{V}{\text{O}}_{{{\text{2max}}}}$: Maximal oxygen uptake
$\dot{V}{\text{O}}_{{{\text{2mean}}}}$: Mean oxygen uptake
